# Hearty recipes for health: the Hakka medicinal soup in Guangdong, China

**DOI:** 10.1186/s13002-022-00502-2

**Published:** 2022-01-25

**Authors:** Mingyan Ding, Shi Shi, Binsheng Luo

**Affiliations:** 1 Shunde Polytechnic, Foshan, 528300 China; 2 South China Limestone Plants Research Center, College of Forestry and Landscape Architecture, South China Agricultural University, Guangzhou, 510642 China; 3 Lushan Botanical Garden, Jiangxi Province and Chinese Academy of Sciences, Lushan, 332900 China

**Keywords:** Hakka, Medicinal food plants, Medicinal soup, Traditional knowledge, Ethnobotany, COVID-19

## Abstract

**Background:**

The Hakka are a subgroup of the Han Chinese, originally from northern China and mainly living in southern China now. Over hundreds of years, the Hakka have developed their own medical practices to prevent and cure diseases, such as medicinal soup, an important characteristic of Hakka cuisine. This study aims at documenting plant taxa used to make Hakka medicinal soup, along with their associated ethnomedical knowledge.

**Methods:**

Data on Hakka soup-making plants were collected through key-informant interviews, semi-structured interviews, participatory field collection, and direct observation. The choosing of participants has followed the snowball sampling method. Herbs used for preparing medicinal soup were purchased on the local market or collected from the wild, and voucher specimens were collected and identified. The study result was also compared with published studies on soup-making plants in other parts of Guangdong province and the Hakka areas in Fujian Province of China.

**Results:**

Eighty-three taxa belonging to 70 genera within 38 families were listed by our informants as being used to treat 55 kinds of health problems. Most documented plants are herbaceous species, followed by shrubs and woody liana. Roots were the most frequently used plant parts for medicinal purposes, followed, in descending order, by the whole plant, rhizomes, and flowers. Most plants used to prepare medicinal soup are wild-harvested (56 species), 4 cultivated, and 23 wild harvested or cultivated. According to the comparison, 18 Hakka medicinal soup species are shown both in Fujian Province and Guangdong Province. The Hakka soup-making plants in both provinces share very similar therapeutic functions. This study helps to extend the Hakka menu in both provinces. The study comparison also showed a big difference in the herb ingredients between Guangdong Hakka medicinal soup and Cantonese slow-cooked soup.

**Conclusion:**

Hakka medicinal soup is an important feature of the Hakka dietary culture. More studies are needed to be undertaken, especially on the efficacy and safety of this medicinal soup. Moreover, cultivation and conservation efforts are required to ensure the sustainability of the species that are used as ingredients in the soup. Consequently, further commercial development of medicinal soup should be promoted.

## Background

The traditional food system has played an important role in daily food consumption and health care worldwide, especially in third-world countries [1]. However, some traditional knowledge about practicing the traditional food system has experienced a threat of disappearing due to fast urbanization and modernization [2]. When the pandemic COVID-19 has attacked the world since the end of 2019, the practice of traditional food systems has re-obtained attention because the indigenous community has shown a series of self-response against the food and medicine shortage during the pandemic time [3]. Therefore, the traditional food system is an important safety buffer providing the indigenous people with natural energy sources and extra nutrition in daily life against the consequences caused by big disasters. The relative traditional knowledge is also worth studying and protecting by the world nowadays.

With the development of the global economy, people have better living conditions, the requirement for food became not just to satisfy the survival needs but also to maintain human health [4]. Thus, the "green", "natural", "medicinal" and "organic" food are pursued by people [5]. The medicinal food plants have thus become a new trend for the food system, which is usually used by indigenous communities for daily health promotion [6]. Since ancient times, China has traditionally used food for health care [7]. For instance, when having a Chinese hotpot ("Huo Guo"), people always like to add different traditional Chinese medicines into the pot for health care purposes [8]. Typically, some people process the medicinal plants into a beverage like tea or medicinal soup, which is very common in the south of China. For example, in the Guangdong Province of China, the culture of cooling herbal tea is extremely popular locally; in the Chaoshan area of Guangdong Province, a study case by Li et al. has reported that more than 186 plant taxa can be used to make herbal tea [9].

As the focus of this paper, the famous diet therapy culture of the Hakka people in China is also a great example of the traditional use of medicinal food plants. The Hakka means "guest families". Hakka people are widely considered to be a subgroup of the Han Chinese, originally migrated from northern China in 300 A.D. to avoid war and natural disasters [10, 11]. According to the statistics, more than 45 million Hakka people live in China [12]. The soup made by medicinal food plants is the most typical dish for Hakka people, consumed during every meal in Hakka households. The Hakka people commonly used meats like pork, chicken, duck, young pigeon, and fish as soup ingredients. The soup is prepared as follows: one kind of meat, along with a single medicinal herb or a combination, is placed in a clay pot, marmite, or stewing pot, covered with water. The contents are brought to a boil and then simmered for 1–3 h to produce the medicinal soup. The soup is often consumed within households but is also supplied to restaurants as a typical Hakka cuisine. With the spread of Hakka restaurants, Hakka medicinal soup is becoming increasingly popular in non-Hakka areas. Combining meat with medicinal herbs is thought to improve the taste. The addition of the meat removes the bitterness of the herbs and adds a delicious flavor to the soup. Furthermore, according to the locals, experience has shown that adding meat to the medicinal soup reduces the likelihood of adverse drug reactions. There have only been a few studies on Hakka medicinal soup until now.

One of our former studies in the Hakka area in Fujian Province has reported 42 plant taxa for medicinal soup, which has played an important role in local people's daily lives [13]. A study by Au et al. revealed that there is also plenty of traditional knowledge about medicinal plants in the Guangdong Hakka area, including medicinal soup consumption [12]. Guangdong Province is one of the main habitats of Hakka people in China, with the biggest Hakka population compared to other provinces in China. According to the field trip, we also found that local Hakkas drink medicinal soup every day. Thus, we hypothesized that (1) the Hakka community in Guangdong Province holds rich knowledge about medicinal soup; (2) the Hakka soup-making plants in Guangdong Province are similar to the west of Fujian Province, another main Hakka habitation in China.

In order to verify our hypothesis, an ethnobotanical study was carried out in the Guangdong Hakka area during 2019–2020, aiming to (1) investigate and record the traditional knowledge about medicinal soup in the Guangdong Hakka area; (2) analyze the inventory of the medicinal soup-making plants in Guangdong Hakka area and compared to the study result in the Fujian Hakka area. Additionally, we would like to discuss what kind of role this knowledge plays in the local community, especially during the COVID-19 pandemic. We hope the findings can contribute to the further protection and the future application of related knowledge about traditional soup-making plants.

## Methods

### Study area

**Fig. 1:**
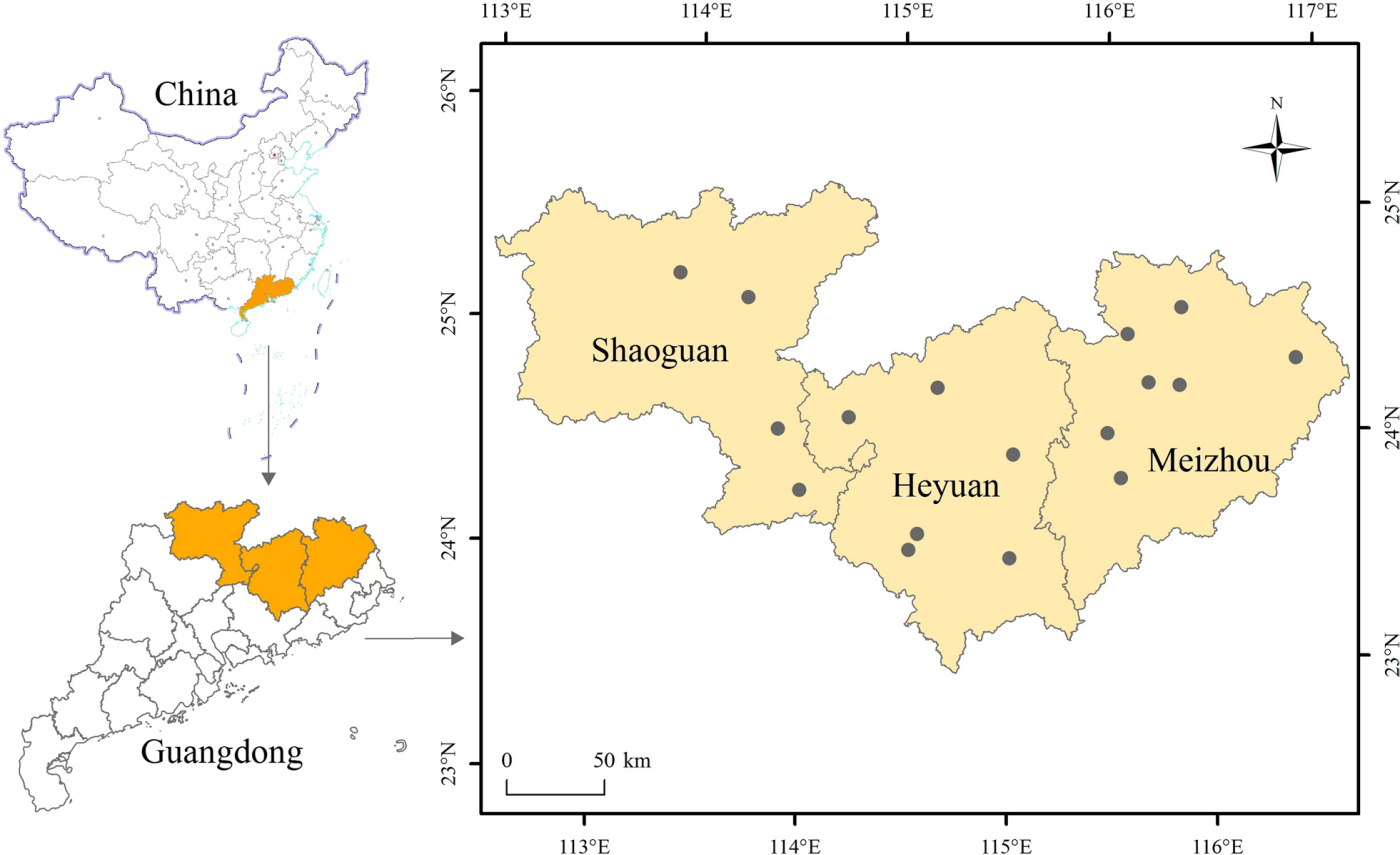
Location of the study area

The study area (Fig. 1), located at 22°26′ N–25°31′ N, 112°50′ E–116°56′ E, encompasses a total area of about 64,800 km^2^ [13, 14]. The region's climate is subtropical, with short, mild, and dry winters and long, hot, and wet summers [14]. Seventeen counties and districts were chosen for the investigation, including Meizhou City (Meijiang District, Meixian County, Jiaoling County, Pingyuan County, Xingning County, Dabu County, and Wuhua County); in Shaoguan City (Xinfeng County, Wengyuan County, Shixing County, and Renhua County); and in Heyuan City (Yuancheng District, Heping County, Lianping County, Longchuan County, Dongyuan County, and Zijin County). "Pure" Hakka communities are predominant in all of these areas (Hakka comprise more than 95% of their populations) [12].

### Data collection

Field surveys were conducted in 2019 and 2020. A total of 213 informants were interviewed, of whom 127 were male and the rest 86 were female. Everyone who participated was informed of the nature and the purpose of the project. Special care was taken in selecting informants. All the informants were Hakka, and only those familiar with medicinal herbs were considered and chosen by the snowball sampling method [15]. The key informant interview method was also used. People who gathered wild herbs prepared medicinal soups, sold herbs (herbal medicinal hawkers, medicinal plant retailers, and e-store operators), and Hakka herbalists were chosen as the key informants. Semi-structured interviews, participatory field collection, and direct observation were used to record the detailed information about the plants used for medicinal soup, including their vernacular names, useful parts, therapeutic function, preparation method, and so on. The questions we asked during the interview included the following: (1) What plant and what part do you use for making soup; (2) Why do you use this species; (3) How do you process it; (4) Where and when do you collect it. Voucher specimens were collected and deposited in the Lushan Botanical Garden. Identification of the plants was followed by *the Flora of China*, *the Flora of Guangdong*, and *the Plant List* (http://www.theplantlist.org/) [16, 17].

The inventory about soup-making plants in this study was also compared quantitatively to the study carried out in the Fujian Hakka area [13]. The similarities, differences between the soup-making plants in two Hakka areas were analyzed.

## Results and discussion

### Plants used as ingredients of Hakka medicinal soup

**Fig. 2:**
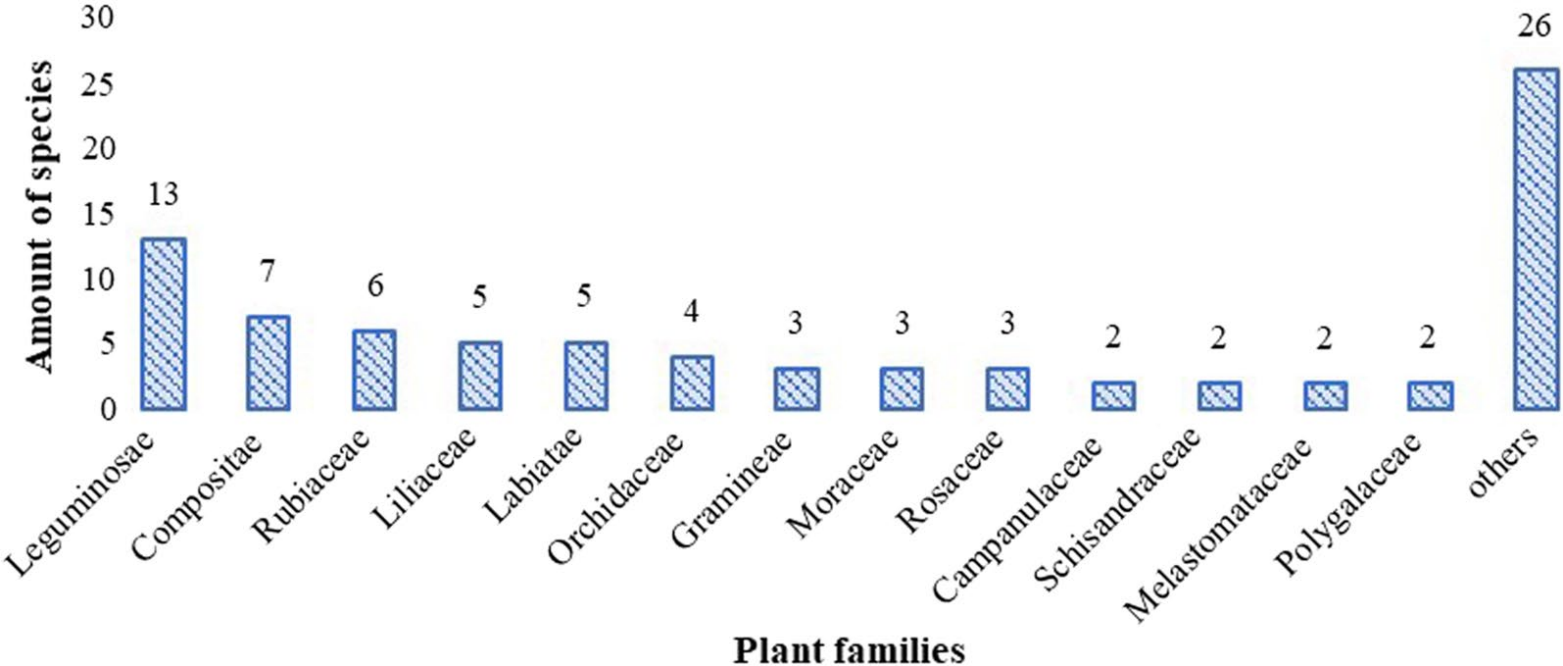
Taxonomic distribution of soup-making plants in the Guangdong Hakka area

Altogether, 83 taxa (including four varieties) used for medicinal soup were listed by our informants. Most of the species are widely distributed, and only seven species are endemic to China, including *Alyxia sinensis* Champ. ex Benth., *Polygala fallax* Hemsl., *Salvia prionitis* Hance, *Schizostachyum dumetorum* (Hance) Munro, and *Taxillus sutchuenensis* (Lecomte) Danser. All taxa recorded belong to 70 genera within 38 families (Table1). Five species were pteridophytes, and the rest were spermatophytes. Leguminosae was the most represented family (13 species), followed by Compositae (7 species) and Rubiaceae (6 species) (Fig. 2). The taxonomic distribution fully indicated the diversity of Hakka soup-making plants in Guangdong Province. The majority of the documented soup-making plants are herbaceous species (40 species, 48.2%), followed by shrubs (14 species, 16.9%) and woody liana (9 species, 10.9%).

**Table 1:** The plants used for making Hakka medicinal soup in Guangdong, China

Latin name	Hakka name	Family	Part of plant	Medical use(s)	**Cultivated/** **wild**	**Dry/** **fresh**	**Voucher ID**
*Abrus pulchellus* subsp. *cantoniensis* (Hance) Verdcourt	Gêgudcau	Leguminosae	Roots, stems and leave	Clear heat and promote diuresis, relieve liver and dissipate stasis	Cultivated and wild	Dry	MZ156
*Abrus pulchellus subsp. mollis (Hance) Verdc*	Gêgudcau	Leguminosae	Roots, stems and leave	Clear heat and promote diuresis, relieve liver and dissipate stasis	Wild	Dry	HY105
*Acorus gramineus* Aiton	Sagcongpu	Acoraceae	Whole plant	Eliminate dampness and stimulate appetite, induce resuscitation and sweep phlegm	Wild	Fresh or dry	SG134
*Adenophora petiolata* subsp. *hunanensis* (Nannfeldt) D. Y. Hong & S. Ge	Sasen	Campanulaceae	Roots	Moisten lung and resolve phlegm, help produce saliva, and slake the thirst	Wild	Dry	SG91
*Adenophora tetraphylla* (Thunb.) Fisch	Sasen	Campanulaceae	Roots	Moisten lung and resolve phlegm, help produce saliva, and slake the thirst	Cultivated and wild	Dry	MZ12
*Agrimonia pilosa* Ledeb	Sanfungcao	Rosaceae	Whole plant	Astringing to arrest bleeding, strengthen the heart	Wild	Dry	HY089
*Alsophila spinulosa* (Wall.ex Hook.) R. M. Tryon	Fitianliung	Cyatheaceae	Stems	Expel wind-damp, stop cough and resolve phlegm	Wild	Dry	SG09
*Alyxia sinensis* Champ.ex Benth	Cgunêntên	Apocynaceae	Roots	Move *qi* and activate blood, expel wind-damp, anesthesia, and analgesia	Wild	Dry	SG51
*Angiopteris fokiensis* Hieron	Mataigiad	Angiopteridaceae	Rhizome and stipes	Clear heat and drain dampness, stop blooding, stop the pain	Wild	Dry	SG182
*Anoectochilus roxburghii* (Wall.) Lindl	Kimyinzi	Orchidaceae	Whole plant	Clear heat, cool blood, dispel dampness, relieve toxicity	Cultivated and wild	Dry	SG89
*Artemisia argyi* H.Lév. & Vaniot	Nê	Compositae	Whole plant	Warm meridians to dispel dampness and cold, stop blooding, anti-inflammation, resolve asthma, stop cough, prevent abortion	Wild	Dry	HY214
*Bauhinia championii* (Benth.) Benth	Giuliungtên	Leguminosae	Roots and older stems	Activate blood and dissipate stasis, activate collaterals, calm the mind and stop the pain	Wild	Dry	HY195
*Bombax ceiba* L	Mugmiên	Bombacaceae	Flowers	Clear heat and eliminate dampness	Cultivated and wild	Dry	MZ60
*Callerya speciosa* (Champion ex Bentham) Schot	Ngiutailid	Leguminosae	Roots	Activate meridians and collaterals, reinforce deficiency and moisten the lung and invigorate the spleen	Wild	Dry	HY167
*Canarium album* (Lour.) DC	Kamlam	Burseraceae	Fruits	Clear heat and relieve toxicity, relieve sore throat, and resolve phlegm, help produce saliva and slake thirst, dispel the effects of alcohol	Cultivated and wild	Dry	HY076
*Celosia argentea* L	Gêgunggifa	Amaranthaceae	Flowers and seeds	Stop blooding, cool blood, stop diarrhea	Cultivated	Dry	HY038
*Chamaecrista mimosoides* (L.) Greene	Tiêngapcau	Leguminosae	Stems and leave	Clear heat and relieve toxicity, anti-inflammation, clear summer-heat and dampness, promote diuresis, dispel the effects of alcohol, remove food stagnation, and reinforce the kidney	Wild	Dry	HY075
*Clerodendrum fortunatum* L	Pagfadênlung	Verbenaceae	Roots	Clear heat and purge fire, anti-inflammation, relieve toxicity, stop cough, and ease pain	Wild	Dry	HY030
*Dendrobium officinale* Kimura et Migo	Sagfu	Orchidaceae	Whole plant	Tonify stomach and help produce saliva, nourish yin and clear heat	Cultivated and wild	Dry	MZ158
*Dendrocalamus latiflorus* Munro	Mazug	Gramineae	Seedling	Clear heat and resolve phlegm, replenish qi and harmonize the stomach	Cultivated and wild	Dry	HY202
*Elephantopus scaber* L	Tizamtêu	Compositae	Whole plant	Clear heat, relieve toxicity, relieve swelling and promote diuresis	Wild	Dry	SG54
*Elephantopus tomentosus* L	Pagboi	Compositae	Whole plant	Clear heat, relieve toxicity, relieve swelling and promote diuresis	Wild	Dry	HY050
*Emilia sonchifolia* (L.) DC. ex DC	Fungboizi	Compositae	Whole plant	Clear heat, promote diuresis, cool blood, relieve toxicity	Wild	Fresh	MZ200
*Eriobotrya japonica* (Thunb.) Lindl	Pipa	Rosaceae	Flowers	Promote diuresis and clear heat, stop thirst and cough	Cultivated	Dry	MZ08
*Litsea cubeba* (Lour.) Pers	Santonggin	Lauraceae	Roots	Nourish kidney, strengthen muscles and bones	Wild	Dry	MZ71
*Ficus hirta* vahl	Ñzimaotao	Moraceae	Roots	Expel wind-damp, replenish qi and strengthen the exterior	Wild	Dry	SG78
*Ficus pandurata* Hance	Ngiunênsuggên	Moraceae	Roots	Nourish and keep-beauty, clear liver and improve vision, expel summer heat	Cultivated and wild	Dry	MZ45
*Flemingia macrophylla* (Willd.) Merr	Yidtiaugên	Leguminosae	Roots	Expel wind-damp, relax tendon and activate collaterals, strengthen sinews and bones, anti-inflammation, and stop the pain	Wild	Dry	HY213
*Flemingia prostrata* Roxb	Yidtiaugên	Leguminosae	Roots	Expel wind-damp, relax tendon and activate collaterals, strengthen sinews and bones, anti-inflammation, and stop the pain	Wild	Dry	SG76
*Gardenia jasminoides* J.Ellis	Giziguong	Rubiaceae	Roots	Clear heat and promote diuresis, decrease internal heat and relieve fidget, cool blood and relieve toxicity, dissipate stasis	Cultivated and wild	Dry	SG90
*Gastrodia elata* Blume	Tiênma	Orchidaceae	Rhizome	Promote intelligence, warm middle-jiao and tonify deficiency	Cultivated	Dry	SG40
*Gynostemma pentaphyllum* (Thunb.) Makino	Nyêsen	Cucurbitaceae	Roots,stems and leave	Anti-inflammation, relieve toxicity, eliminate phlegm and stop cough	Cultivated and wild	Dry	SG174
*Hedyotis chrysotricha* (Palib.) Merr	Ngibtingung	Rubiaceae	Roots	Clear heat and resolve phlegm, dispel stasis to promote regeneration, detox snake venom	Wild	Dry	HY219
*Hedyotis diffusa* Willd	Pagfasasadcau	Rubiaceae	Whole plant	Clear heat and relieve toxicity, active blood and promote diuresis, remove stagnation, and stop the pain, anti-cancer	wild	Fresh or dry	HY184
*Hemerocallis citrina* Baroni	Gimzemcoi	Liliaceae	Flowers	Invigorate stomach, promote diuresis, relieve swelling	Cultivated and wild	Dry	SG47
*Houttuynia cordata* Thunb	Yuxingcao	Saururaceae	Whole plant	Clear heat, relieve toxicity, promote diuresis	Wild	Dry	SG87
*Hylocereus undatus* (Haw.) Britton & Rose	Jiamfa	Cactaceae	Flowers	Clear heat, moisten the lung	Cultivated and wild	Dry and fresh	HY079
*Imperata cylindrica* (L.) Raeusch	Pagmaogoung	Poaceae	Rhizome	Clear heat and promote diuresis	Wild	Dry	MZ30
*Duhaldea cappa* (Buchanan-Hamilton ex D. Don) Pruski & Anderberg	Yongngigug	Compositae	Whole plant	Resolve phlegm and resolve asthma, activate blood and regulate menstruation	Wild	Dry	HY175
*Juncus effusus* L	Dênximcau	Juncaceae	White pitch of the stems	Promote diuresis, cool and tranquilize	Wild	Dry	HY112
*Kadsura coccinea* (Lem.) A.C.Sm	Hiongtênzi	Schisandraceae	Roots	Move qi and activate blood, relieve swelling and pain, expel wind-damp	Wild	Dry	HY164
*Kadsura heteroclita* (Roxb.) Craib	Hiongtênzi	Schisandraceae	Roots	Move qi and activate blood, relieve swelling and pain, expel wind-damp	Wild	Dry	SG30
*Lablab purpureus* (L.) Sweet	Biêntêu	Leguminosae	Fruits	Dispel summer heat and dampness, invigorate the spleen and stop diarrhea	Cultivated	Dry	SG34
*Laggera alata* Nanth	Liugngigug	Compositae	Whole plant	Expel wind-damp, clear heat and moisten dryness	Wild	Dry and fresh	SG103
*Leonurus Artemisia* (Lour.) S. Y. Hu	Cunnê	Labiatae	Whole plant	Dispel stasis to promote regeneration, activate blood and regulate menstruation	Wild	Dry	HY106
*Lespedeza cuneata* (Dum.Cours.) G.Don	Gonjincau	Leguminosae	Roots	Invigorate spleen, remove food stagnation, remove infantile malnutrition	Wild	Dry	HY166
*Leonurus japonicus* Houtt	Pagfacunê	Labiatae	Whole plant	Dispel stasis to promote regeneration, activate blood and regulate menstruation	Wild	Dry	SG79
*Lycium chinense* Mill	Tigudpi	Solanaceae	Roots and fruits	Cool blood and clear heat, clear lung and decrease internal heat	Cultivated and wild	Dry	MZ88
*Lysimachia fortunei* Maxim	Caggiogcau	Primulaceae	Whole plant	Clear heat and drain dampness, activate blood and regulate menstruation	Wild	Dry	HY101
*Maclura cochinchinensis* (Lour.) Corner	Conposag	Moraceae	Roots	Stop cough and resolve phlegm, expel wind-damp	wild	Dry	MZ99
*Mallotus apelta* (Lour.) Müll.Arg	Pagmaodang	Euphorbiaceae	Roots	clear heat and dispel dampness, induce astringency and dispel stasis	Cultivated and wild	Dry	HY109
*Melastoma dodecandrum* Lour	Aigiogngian	Melastomataceae	Roots	Relax tendon and activate blood, enrich the blood and prevent abortion	wild	Dry	MZ109
*Morinda officinalis* F.C.How	Gêconggin	Rubiaceae	Roots	Nourish kidney, strengthen muscles and bones, expel wind-damp	Cultivated and wild	Dry	HY222
*Nephrolepis cordifolia* (L.) C. Presl	Sagvongpi	Nephrolepidaceae	Tuber	Clear heat and drain dampness, moisten the lung and stop cough, remove food stagnation	Cultivated and wild	Fresh	HY165
*Ocimum basilicum* L	Giuqiêncha	Labiatae	Whole plant	Expel wind and release superficies, dispel the wind and relieve swelling, dispel stasis, and stop the pain	Cultivated and wild	Dry	MZ68
*Ophiopogon japonicus* (Thunb.) Ker Gawl	Magdung	Liliacae	Root tuber	Help produce saliva and slake thirst, moisten the lung and stop cough	Cultivated and wild	Dry	HY194
*Paederia foetida* L	Gêsitên	Rubiaceae	Overground part	Expel wind-damp, remove food stagnation	Wild	Dry	HY116
*Patrinia villosa* Juss	Fuzai	Valerianaceae	Whole plant	Clear heat, relieve toxicity, relieve swelling, activate blood and dispel stasis	Wild	Dry	SG123
*Peristrophe bivalvis* (L.) Merr	Fungsixiên	Acanthaceae	Stems and leave	Anti-inflammation and promote diuresis, clear heat and relieve toxicity, clear lung, and stop cough	Cultivated and wild	Dry	HY149
*Pholidota chinensis* Lindl	Sagfulu	Orchidaceae	Whole plant	Nourish yin, clear lung, drain dampness, dissipate stasis	Wild	Dry and fresh	HY205
*Phymatopteris hastata* (Thunb.) Pic. Serm	Gimgêgiog	Polypodiaceae	Whole plant	Dispel the wind and clear heat, drain dampness, relieve toxicity	Wild	Dry	MZ84
*Polygala chinensis* L	Ziboiginngiu	Polygalaceae	Stems and leave	Stop cough, remove food stagnation, activate blood and dissipate stasis	Wild	Dry	HY084
*Polygala fallax* Hemsl	Daosuiwong	Polygalaceae	Roots	Strengthen spleen and kidney, nourish *yin* and purge fire	Wild	Dry	MZ108
*Psychotria serpens* L	Congêntên	Rubiaceae	Whole plant	Relax tendon and activate collaterals, strengthen muscles and bones, dispel the wind and stop the pain, cool blood, and relieve swelling	Wild	Dry	HY150
*Pteridium aquilinum* var. *latiusculum* (Desv.) Underw. ex A. Heller	Giad	Pteridiaceae	Leave	Promote diuresis and promote diuresis, clear heat and relieve toxicity	Cultivated and wild	Dry	HY168
*Pueraria montana* (Lour.) Merr	Godgiong	Leguminosae	Roots	Release superficies and clear heat, help produce saliva and slake thirst, stop diarrhea	Wild	Dry	HY128
*Pueraria montana* var. *lobata* (Willd.) Sanjappa & Pradeep	Godgiong	Leguminosae	Roots	Release superficies and clear heat, help produce saliva and slake thirst, stop diarrhea	Wild	Dry	MZ140
*Isodon serra* (Maxim.) Kudô	Haivongcau	Labiatae	Stems and leave	clear heat and drain dampness, cool blood and dispel stasis	Wild	Dry	SG99
*Rhodomyrtus tomentosa* (Aiton) Hassk	Dongli	Myrtaceae	Roots	Nourish blood, activate blood and dredging collaterals, astringe to arrest diarrhea	Wild	Dry	SG143
*Rhus chinensis* Mill	Yamsonkua	Anacardiaceae	Roots	Expel wind-damp, dissipate stasis, clear fever and relieve toxicity	wild	Dry	HY046
*Rosa Laevigata* Michx	Tonggonzi	Rosaceae	Roots and fruits	Activate blood and dissipate stasis, expel wind-damp, relieve toxicity and astringe	Wild	Dry	SG188
*Salvia prionitis* Hance	Funggêncau	Labiatae	Whole plant	Moisten lung and stop cough, anti-inflammation and anti-bacteria, relieve sore throat	Wild	Dry	HY039
*Schizostachyum dumetorum* (Hance) Munro	Miauzug	Gramineae	Seedling	Clear heat and relieve toxicity, promote appetite	Cultivated and wild	Dry	HY156
*Smilax china* L	Ngangfantêu	Liliaceae	Rhizome	Clear heat and relieve toxicity, eliminate dampness and relieve swelling	wild	Dry	HY130
*Smilax glabra* Roxb	Ngangfantêu	Liliaceae	Rhizome	Clear damp-heat, relieve toxicity, invigorate spleen and stomach	Cultivated and wild	Dry	MZ131
*Smilax riparia* A. DC	Ngiumigiad	Liliaceae	Rhizome	Dispel the wind and activate collaterals, eliminate phlegm and stop cough	Wild	Dry	SG86
*Sonerila cantonensis* Stapf	Fungdêucau	Melastomataceae	Whole plant	Clear heat and relieve toxicity, promote diuresis	Wild	Dry	HY218
*Tadehagi triquetrum* (L.) H.Ohashi	Gêusadle	Leguminosae	Roots	clear heat and relieve toxicity, invigorate the spleen and promote diuresis	Wild	Dry	MZ17
*Taxillus sutchuenensis* (Lecomte) Danser	Gisangê	Loranthaceae	Whole plant	Nourish liver and kidney, strengthen muscles and bones, expel wind-damp; nourish the blood and prevent abortion	Wild	Dry	HY124
*Triumfetta rhomboidea* Jacq	Vongfasênmatêu	Tiliaceae	Whole plant	Release superficies and clear heat, promote diuresis and remove stagnation	Wild	Dry	HY064
*Uraria crinita* (L.) DC	Dagsengên	Leguminosae	Roots	Dissipate stasis and stop blooding, clear heat and stop cough	Cultivated and wild	Dry	HY035
*Vernonia cinerea* (L.) Less	Yahiongngiu	Compositae	Whole plant	Expel wind and clear heat, draw out the poison and relieve swelling, calm the mind and nerves, remove food stagnation	Wild	Dry	MZ78
*Wikstroemia indica* (L.) C.A. Mey	Timiêngin	Thymelaeaceae	Roots and stems	Clear heat, relieve toxicity, relieve swelling and pain	Wild	Dry	SG102

**Fig. 3:**
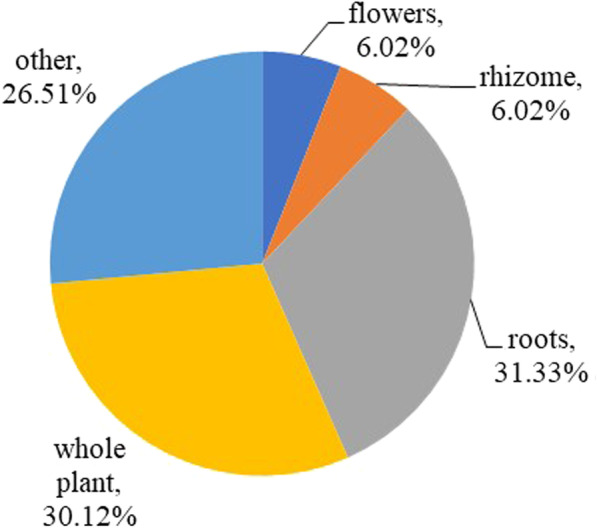
Plant parts used by the Guangdong Hakka for preparing medicinal soup

The medicinal parts used for soup making are shown in Fig. 3. Roots (26 species, 31.3%) were the most frequently used plant part for medicinal purposes, followed, in descending order, by the whole plant (25 species, 30.1%), rhizomes (5 species, 6.0%), and flowers (4 species, 4.8%). The vast majority of the plants are dried (76 species) because it is easier to restore in a long period in such a humid and warm environment; more importantly, dry plants can somehow reduce the strong original flavor and make the locals easier to accept. For example, the *Houttuynia cordata* is one of the most popular food in the Southwest of China, where the locals usually eat its root freshly, whose flavor is too strong for a lot of outsiders [18]. However, the dry *Houttuynia cordata*, consumed by Hakka, has a much less strong flavor, not covering other ingredients' taste.

Most plants used to prepare medicinal soup are wild-harvested (56 species), four species are cultivated, and 23 are either wild-harvested or cultivated. Thus, more than half of these taxa cannot be cultivated. The wild-harvested plants are collected from forests, hillsides, roadsides, or wastelands nearby the communities.

### Medical functions of herbs used in Hakka medicinal soup

**Fig. 4:**
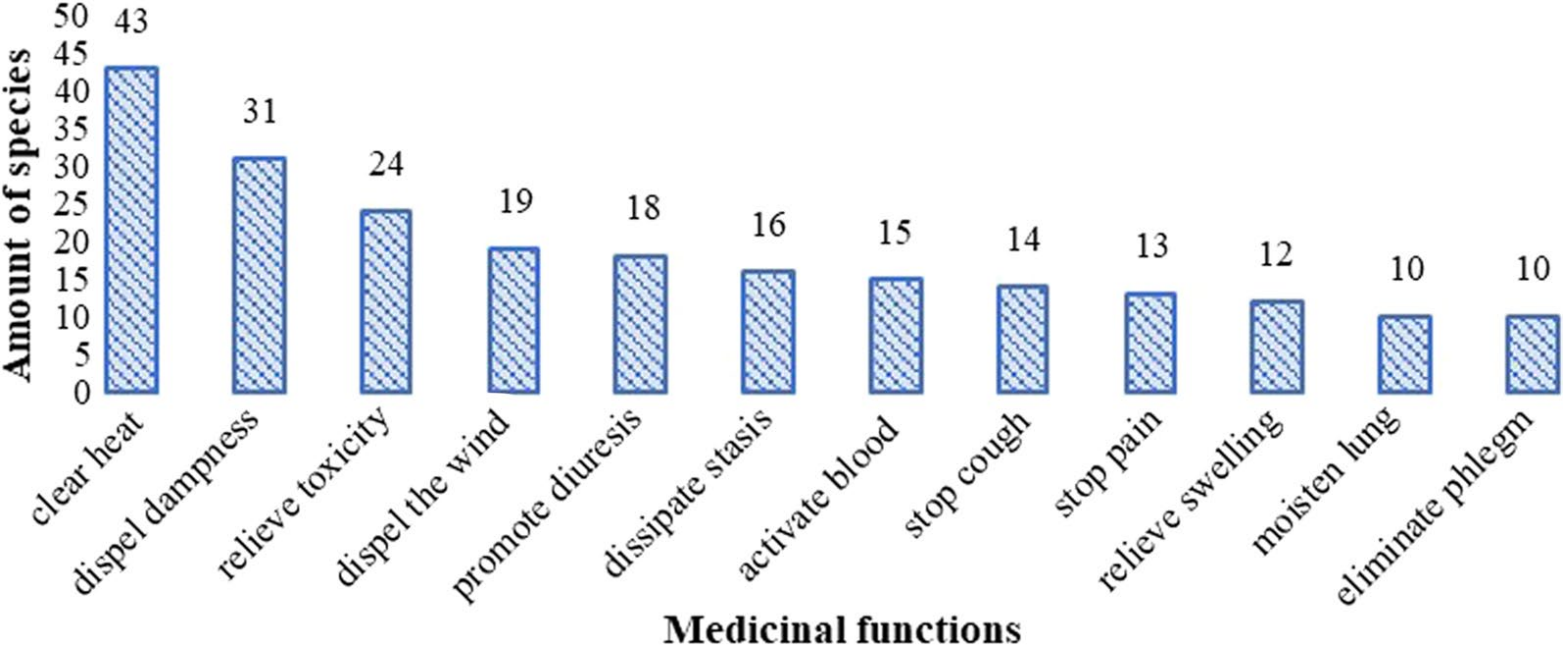
Frequency (> 10) of mentions of medicinal function category

The 83 species for medicinal soup are used to treat 55 kinds of health problems. The functions of the herbs used in the medicinal soup are shown in Fig. 4 (category/frequency > 10). Most of these are used for disease prevention or treatment of ailments. The most common uses recorded in this study, in descending order, were: clearing heat (43 species), dispelling dampness (31 species), relieving toxicity (24 species), and dispelling the wind (19 species). These medicinal functions are closely related to the local natural environment and local Hakka livelihood. The Hakka medical system is closely similar to the traditional Chinese medicine system because historically, as a branch of Han people, Hakka people migrated from the main habitat of Han people at that time. The "heat" does not just mean the "high fever" or "high body temperature", and it is more like an unbalanced of the inner energy. Additionally, the "toxicity" we mentioned here is not just from animals like poisonous insects or snakes; it is the unbalance or unhealthy substance accumulation caused by unusual factors like extreme weather, miasma, etc. Local areas are in mountainous topography associated with the very heavy miasma and humid subtropical climate [13, 14]. Thus, the medicinal soup for clearing inner heat and detoxication is popular locally. Local livelihoods are mostly practicing agriculture, which is heavy physical labor. In such a humid mountainous area, rheumatism is frequently happened, which is related to the "dampness" and "wind" mentioned above. Thus, clearing dampness and clearing wind are also popular locally.

In addition, some of the herbal ingredients of the medicinal soup have specialized functions that apply to particular categories of people. For example, according to the locals, *Artemisia argyi*, *Melastoma dodecandrum*, and *Taxillus sutchuenensis* are used to prevent pregnant women from aborting. Preparations of *Lespedeza cuneata*, and *Uraria crinita* are used to treat infantile malnutrition.

Before introducing modern medicine, some medicinal soups were important mainstream remedies for different health conditions locally; some are still popular [19]. There is scientific evidence that some of the components of chicken soup inhibit neutrophil migration [20]. This biological reaction may be linked to an anti-inflammatory effect that could hypothetically lead to a temporary easing of symptoms of illness [20]. However, in the case of Hakka medicinal soup, few studies have been conducted on its pharmacological efficacy, let alone its clinical applications. The related findings focus on the phytochemicals of the herbs used for Hakka medicinal soup and their efficacy. However, some folk used edible plants that could be toxic or chronically toxic. Controlled studies should be undertaken to determine the bioactivity and toxicity of Hakka medicinal soup, the active constituents of the herbs, and their mechanism of function, both in vitro and in vivo. With the Hakka medicinal soups getting more popular, food safety studies are needed urgently.

### Comparison with surrounding areas

Guangdong Hakka region is geographically close to the Fujian Hakka region, and they both share almost the same climate and floras [10]. Consequently, the Hakka communities in both regions share a very similar culture and even the same origin [10]. Thus, we compared this study with our former one in the Fujian Hakka area, which used the same method and was published in 2019 [13].

**Fig. 5:**
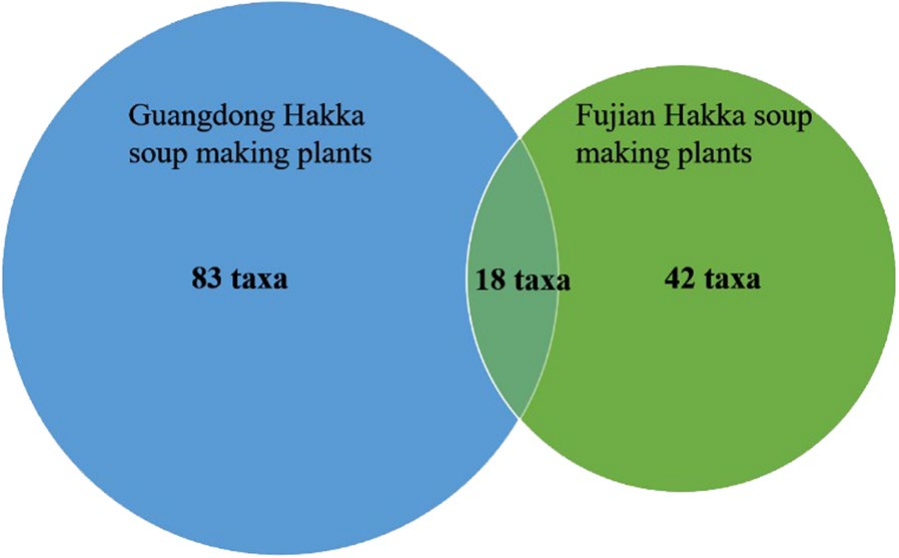
Comparison of soup-making plants in Hakka areas in Guangdong and Fujian Province

According to the comparison, as shown in Fig. 5, both studies share18 species of soup-making plants and have the same usages in both areas due to the medicinal properties and the similar Hakka culture. However, 65 soup-making plants were not mentioned in the study on the Fujian Hakka area, which also indicated the differences in both Hakka areas. The differences could be due to the mountainous topography that cut off the communication between both areas in history. The vernacular names for soup-making plants also show many differences between the two studies. The dialects in two close counties developed some differences even though mostly similar because the traffic is difficult locally in the history.

On the other hand, the amount of Guangdong Hakka soup-making plants is much more than in Fujian Province, which could be due to the impact of other medicinal soup cultures in Guangdong Province. Not just in the Hakka area, medicinal soup is also popular in other parts of Guangdong province, like the Chaoshan area.

Even though the soup-making plant taxa in both Fujian and Guangdong areas showed many differences, we still find, in the level of medicinal function, both soup-making plant inventories share almost the same quality: clearing heat, dispelling dampness, relieving toxicity, and dispelling the wind. The similarity fully indicates that both Hakka areas share very similar environments, climates, and daily livelihoods. However, a similar environment means similar plant distributions. Additionally, except for the endangered species, the plant inventories on both Hakka areas are all ubiquitous species. Thus, the study result can help broaden Hakka cuisine's daily menu in both Hakka areas.

**Fig. 6:**
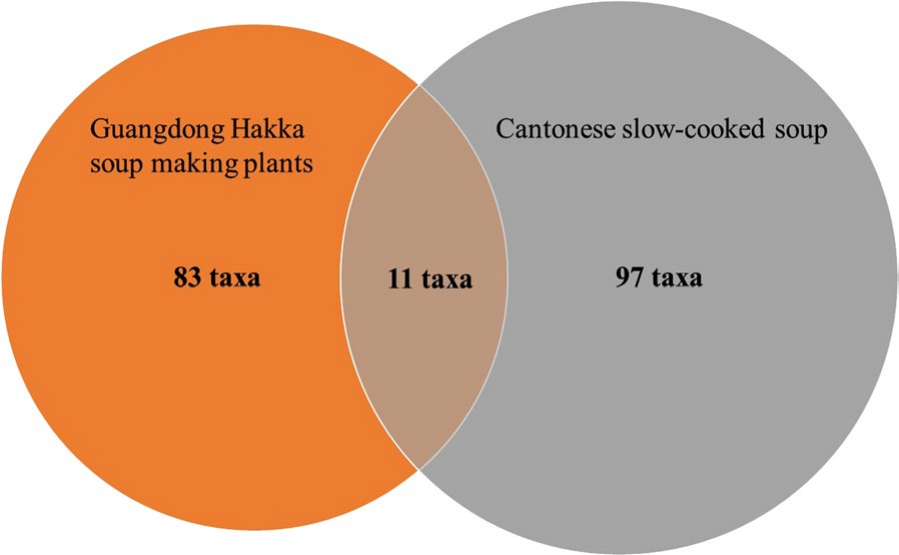
Comparison between Guangdong Hakka soup and CSCS

"Lao Huo Liang Tang", which means Cantonese slow-cooked soup (CSCS), is famous in Cantonese cuisine and culture. Although CSCS is popular in Guangdong Province, it is not a Hakka cuisine. Liu et el has systematically studied the CSCS using ethnobotanical methods [21]. We also compared our inventory with the one published by Liu et al. [21] (Fig. 6). As a result, both studies only share 11 species in common. Additionally, the CSCS aims for a different medicinal purpose, like around 30 species are used for tonifying Qi or tonifying Yan, by following the classic traditional Chinese Medicine (TCM) theory. Also, CSCS uses more commercialized plant ingredients or the ones common in TCM like ginseng, *Amomum villosum*, *Eucommia ulmoides*, etc. The areas that consume slow-cooked soup in Guangdong are more developed, while the Hakka communities are in mountain areas with less developed economies and transportation. The comparison indicates a very big difference between Cantonese culture and Hakka culture.

### Commercialization and sustainability of Hakka medicinal soup

According to our findings, some medicinal species are under threat as a result of modernization and industrialization processes, leading to habitat destruction and excessive exploitation. For example, the IUCN Red List of Threatened Species lists *Pholidota chinensis* as near threatened (NT), *Gastrodia elata* as vulnerable (VU), and *Dendrobium officinale* as critically endangered (CR). In the China Species Red List, *Callerya speciosa* (Champion ex Bentham) Schot and *Kadsura coccinea* are listed as VU; *Alsophila spinulosa* is listed as NT, and *Anoectochilus roxburghii* is listed as endangered (EN) [22]

According to the research, raw soup-making plants are sold in local wet markets, herbal stores, supermarkets, and e-stores online, with little or no packaging. Both local people and tourists like to consume herbs for preparing medicinal soup. With the development of e-commerce and the spread of Hakka restaurants all over China, soup-making herbs are consequently in demand in areas outside of Hakka communities. Given the continuously increasing demands for soup-making plants, more and more plant species have been cultivated locally. Cultivation of medicinal plants conserves plant resources and makes their collection convenient and accurate [23]. Examples include *Ficus hirta*, *Gardenia jasminoides*, and *Rabdosia lophanthoides*, cultivated in good agricultural practice (GAP) farms in Yuancheng District, Fengshun County, and Pingyuan County, respectively. Other commonly used herbs previously harvested from the wild, such as *Anoectochilus roxburghii*, *Smilax glabra* and *Uraria crinita* are now being cultivated according to the field investigation. However, the threatened status of wild herbs does not give rise to optimism because the domestication of herbs through farming is not always technically possible. We found that Alsophila spinulosa, a tree fern with a long-life cycle, is under serious threat and very difficult to cultivate during our survey. Cultivated plants are also sometimes considered qualitatively inferior compared to wild ones locally. For instance, wild, dried *Anoectochilus roxburghii* plants (9,000–28,000 ¥/kg) are considerably more expensive than the cultivated variety (1,250–6,000 ¥/kg). Consequently, they are over-exploited and threatened. The over-exploited phenomenon of *Anoectochilus roxburghii* is also mentioned in the Hakka area in West Fujian Province. The local supervision department should pay attention to the trading and the protection of those endangered wild plants.

### The medicinal food plants during the COVID-19 pandemic

As we all know, the whole world has been seriously affected by the COVID-19 epidemic since the end of 2019. Although some remote areas have not been attacked directly, they were still facing a series of food and medicine shortages because of the breaking down of the normal productions and commerce logistics. However, some self-adjustments have been observed in indigenous communities. The famous ethnobiologist Andrea Pieroni once mentioned: in many cultures, self-made plant-sourced food or beverage has played a very important role in local communities to treat some ailments, contagions, or chronic diseases [24]. In 2020, Pieroni et al. had studied the reactions to the COVID-19 in 17 indigenous communities, and some of the local families seemed to start using some herbal medicine or functional food for treating a respiratory disease or flu [3].

In some cases, the local community increased the consumption of onion, garlic, lemon, and turmeric because the local people believe this plant-sourced food can help resist the virus and boost immunity [3]. However, this is not an isolated phenomenon. In this area, we also observed that some families increased the consumption of Houttuynia cordata, which was believed to be a promising anti-virus medicinal plant locally. In our another study in 2020 (unpublished), many peasant workers had to stay at home because the COVID-19 stopped the operation of factories in cities. Thus, they must go to the wild collecting edible and medicinal plants to maintain their daily consumption.

The phenomenon we observed and mentioned by Pieroni is worth thinking about, which indicated that, in front of natural disasters like big pandemics, some remote communities have their own defensive response. This self-response needs to base on rich biodiversity and traditional knowledge about wild resources utilization. The benefits of this response are not just from the medicinal or edible value of the wild resources but also can help boost people emotionally and psychologically [3]. The pandemic fully addresses the importance of traditional knowledge of plant use and wild resources management.

## Conclusion

Hakka medicinal soup is a very important feature of the Hakka dietary culture, not only to achieve satiety but also to safeguard the health and treat ailments. Eighty-three taxa of medicinal soup-making plants in Hakka areas in Guangdong Provinces have been recorded in this study, which is diverse and fully indicates the local wisdom of adapting to the environment. As it is becoming increasingly popular, both in Hakka and non-Hakka areas, more studies are needed on the efficacy and safety of this medicinal soup, and more attention should be paid to the conservation and cultivation of the plants used. Community-based responses during the pandemics like COVID-19 are also worth studying, bringing more benefits to the locals and providing references for policy-making.

## Data Availability

All data generated or analyzed during this study are included in this published article.
